# Incidental Findings on Brain MRI in People with HIV Infection

**DOI:** 10.1038/s41598-020-66443-6

**Published:** 2020-06-11

**Authors:** Kevin F. Hanna, Harlan R. Sayles, Jennifer O’Neill, Matthew L. White, Tony W. Wilson, Susan Swindells

**Affiliations:** 1 College of Medicine, University of Nebraska Medical Center, Omaha, NE USA; 2 Department of Biostatistics, University of Nebraska Medical Center, Omaha, NE USA; 3 Department of Internal Medicine, University of Nebraska Medical Center, Omaha, NE USA; 4 Department of Radiology, University of Nebraska Medical Center, Omaha, NE USA; 5 Department of Neurological Sciences, University of Nebraska Medical Center, Omaha, NE USA

**Keywords:** HIV infections, Diseases of the nervous system, Magnetic resonance imaging

## Abstract

Background: Incidental findings are a well-known complication of imaging studies done for both diagnostic and research purposes. Little is known about the rates and types of incidental findings found on brain MRI in patients with HIV infection, who may be at risk for HIV-Associated Neurocognitive Disorders (HAND). Methods: The parent study included 108 adults with HIV infection and 125 demographically-matched uninfected controls who completed MRI and neuropsychological testing. Incidental findings were classified by the study team as vascular, neoplastic, congenital, other neurologic, or non-neurologic. Categorical measures were compared using Pearson chi-square tests; continuous measures were compared using t-tests. Results: Among participants with HIV infection, 36/108 (33%) had incidental findings compared to 33/125 (26%) controls (p = 0.248). Rates of incidental findings were significantly correlated with increasing age in both participants with HIV infection (p = 0.013) and controls (p = 0.022). We found no correlation between presence of incidental findings and sex or race/ethnicity among either cohort, and no correlation with CD4 count or HAND status for the HIV-infected cohort. Conclusions: Incidental findings were common in both participants with HIV infection and controls, at higher rates than previously reported in healthy populations. There was no significant difference in prevalence between the groups.

## Introduction

MRI of the brain is increasingly used both for scientific research and as a diagnostic tool to guide clinical medicine. In recent years, new generations of MRI machines have become available to investigators and clinicians providing the ability to achieve higher resolution and greater imaging power. Because of this increase in imaging ability, investigators are better able to detect changes in neuroanatomy and function. With this tendency towards higher processing power, however, has come increasing incidence of incidental findings, both in imaging studies performed for research purposes and for clinical care^1–3^. Incidental findings are defined as “a finding concerning an individual research participant that has potential health or reproductive importance and is discovered during conducting research but is beyond the aims of the study”^2^. Recent studies have found that incidental findings are found on up to 48% of structural MRI studies^3,4^. These incidental findings pose an ethical dilemma, as they often require further workup and diagnostic evaluation, resulting in increased patient anxiety, and increased cost to healthcare systems. Furthermore, many of the subjects in these studies are volunteers and the images obtained in these studies may not be reviewed by a suitably qualified Diagnostic Radiologist; therefore, incidental findings may go unrecognized or the subjects are lost to follow-up^5^. Risks of false-positive findings and the burden of medical follow-up are an ongoing source of discussion in effort to find the best practice for managing such findings^6^.

Previous studies have investigated and characterized incidental findings in the general population, but few studies have applied these methods to specialized patient populations such as those with HIV infection^3,7–9^. HIV is known to cause a wide array of functional neurocognitive changes over time, collectively referred to as HIV-Associated Neurocognitive Disorders (HAND). Although severe neurocognitive impairment has become rare over the last decade, HAND remains a prominent area of research as scientists and clinicians attempt to understand the effects of chronic HIV infection on the brain^10^. To date, there has been only one study that has reported rates of incidental findings on brain MRI in PWH or characterization of those findings^3^.

An ongoing NIH-funded study (MH103220) of magnetoencephalography (MEG) and MRI Markers of HIV-Associated Neurocognitive Disorders Across the Lifespan was designed to examine the brain basis of HAND and identify markers of disease progression^11,12^. In this study, we focus on 108 PWH and 125 matched controls who completed neuropsychological testing, MEG, and brain MRI. The investigative team noted a large number of incidental findings during the conduct of the study and conducted the post-hoc analyses reported here. We hypothesized that PWH have higher prevalence of incidental findings on brain MRI, and that this higher prevalence will be correlated inversely with CD4 T-cell counts.

## Methods

### Study design and participants

The study included 108 adults with HIV-infection and 125 demographically-matched, uninfected controls without cognitive impairment. The controls were matched on age, sex, ethnicity, and handedness. Exclusion criteria for both groups included any active, severe psychiatric illness, drug or alcohol abuse, major neurologic disease or history of significant head trauma. Demographic and medical history data were collected through participant interview and from the medical records. All participants completed neuropsychological tests to assess multiple domains most affected by HIV disease, including: (1) Wide Range Achievement Test (WRAT-4 Reading), (2) Hopkins Verbal Learning Test-Revised, (3) Trailmaking A and B, (4) Grooved Pegboard – dominant and non-dominant, (5) Wechsler Adult Intelligence Scale (WAIS-III) Digit Symbol, (6) WAIS-III Symbol Search, (7) Stroop Interference Task, and (8) Verbal Fluency (letter and category). All participants also completed the self-reported assessment of Activities of Daily Living Scale (ADLS). Composite scores for each domain were computed by calculating demographically-normalized z-scores and taking an average of the z-scores for all tests within that domain. Together with the assessment of activities of daily living, these scores were used to diagnose HAND according to the Frascati guidelines^13^.

Participants with HIV infection underwent CD4 count and viral load testing, and control participants underwent HIV testing to exclude HIV infection. Urine pregnancy testing was performed, if indicated. All participants then underwent MEG using a 306-sensor Elekta MEG system and MRI on a Philips 3-Tesla MRI scanner. The MRI protocol included a structural MRI T1 scan designed to highlight the gray/white matter boundary, a T2-weighted FLAIR (fluid-attenuated inversion recovery) sequence, followed by a diffusion-weighted sequence, and a resting-state fMRI scan. The protocol did not involve contrast agents or sedation of any kind and earplugs were provided to minimize the loud noise from the scanner. In this study, we focus on the T1-weighted structural images. Briefly, participants underwent high-resolution T1-weighted MRI on a Philips Achieva 3 T X-series scanner using an eight-channel head coil and a 3D fast-field echo sequence (TR: 8.09 ms; TE: 3.7 ms; field of view: 240 mm; slice thickness: 1.0 mm with no gap; in‐plane resolution: 1.0 × 1.0 mm).

Each MRI study was read by the same expert neuroradiologist, who was blind to the participant’s HIV status. Incidental findings were classified by the study team as vascular, neoplastic, congenital, other neurologic, or non-neurologic. The diagnoses were made based on the characteristics of the MRI findings.

### Standard protocol approvals, registrations, and patient consents

The Institutional Review Board of the University of Nebraska Medical Center approved this study. Each participant provided written informed consent following a detailed explanation of the study. All participants completed the same protocol.

### Statistical analysis

Categorical measures were compared using Pearson chi-square tests or Fisher’s exact tests while continuous measures were compared using t-tests. All analyses were conducted using STATA v14.2 (The StataCorp, LLC, College Station, TX). P values of less than 0.05 were considered significant.

## Results

### Participants

Participant characteristics are shown in Table 1. The age range was 26–72 years. On average, for the whole cohort, 43% were female and 60% white. There were no significant differences between the groups.

**Table 1:** Participant Characteristics.

	Participants with HIV (%).	Participants without HIV (%)
Age in years (range)	26–72	23–72
Sex		
Male	63 (58)	69 (55)
Female	45 (42)	56 (45)
Race:		
White	63 (58)	76 (61)
Black	31 (29)	34 (27)
Other	14 (13)	15 (12)
Ethnicity		
Non-Hispanic	99 (92)	117 (94)
Hispanic	9 (8)	8 (6)

### Prevalence and types of incidental findings

36/108 (33%) PWH and 33/125 (26%) of control participants exhibited an incidental finding on brain MRI. However, this difference was not statistically significant (p = 0.248). Table 2 describes the 43 incidental findings reported in the PWH cohort and the 43 findings reported among the control arm. Of these, neurologic incidental findings comprised 63% of the findings in the cohort with HIV infection and 44% in the control cohort Vascular findings comprised 10% in the cohort with HIV infection, and 28% in the controls. Neoplastic findings comprised 5% of the findings in both cohorts.. None of these differences were statistically significant between the groups.

**Table 2:** Incidental Findings in Participants with HIV and Controls.

Finding	Participants with HIV Infection (%)	Control Participants (%)
Number with Incidental Findings (%)	36/108 (33%)	33/125 (26%)
Neurologic	27 (62.8)	19 (44.2)
White Matter Loss	20 (46.5)	9 (20.9)
Empty Sella/Pseudotumor Cerebri	6 (14)	8 (18.6)
Basal Ganglia Disease	1 (2.3)	0
Normal Pressure Hydrocephalus	0	1 (2.3)
Chiari Malformation	0	1 (2.3)
Vascular	6 (14)	12 (27.9)
Chronic Small Vessel Ischemic Disease	5 (11.6)	10 (23.3)
Lacunar Lesion	1 (2.3)	1 (2.3)
Brainstem Lesion	0	1 (2.3)
Neoplastic	2 (4.6)	2 (4.6)
Meningioma	1 (2.3)	0
Colloid Cyst	1 (2.3)	0
Pituitary Adenoma	0	1 (2.3)
Interventricular Nodule	0	1 (2.3)
Non-Neurologic	8 (18.6)	10 (23.3)
Maxillary Sinus Disease	6 (14)	7 (16.3)
Ethmoid Sinus Disease	1 (2.3)	1 (2.3)
C3–4 Disc Protrusion with Mild Cord Compression	1 (2.3)	0
Expanded Spinal Cord Canal	0	1 (2.3)
Congenitally Short Pedicles in Upper Cervical Spine	0	1 (2.3)

Note: some participants had more than one finding.

**Figure 1:**
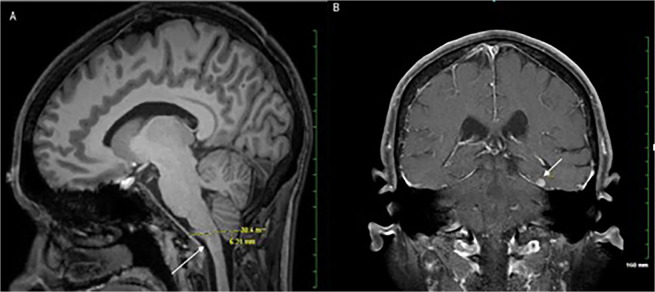
Representative Incidental Findings on Brain MRI. Arrows indicate the abnormalities in each image. Panel (A) shows low lying cerebellar tonsils (type I Chiari malformation) more than 5 mm below the level of the foramen magnum on a T1-weighted sagittal image obtained for diagnostic purposes after the original research image. A meningioma is shown on the T1-weighted coronal image in Panel (B).

Non-neurologic findings comprised 19% of the findings in the cohort with HIV infection and 23% in the controls. Among PWH, the most common incidental findings were white matter loss (47%), empty sella/pseudotumor cerebri (14%), and maxillary sinus disease (14%). Findings of particular clinical significance were a Type I Chiari Malformation found in one of the control patients and a meningioma found in one of the PWH, as shown in Fig. 1.

### Associations between clinical variables and incidental findings in participants with HIV-Infection

Tables 2 and 3 describe how rates of incidental findings were significantly correlated with increased age in both PWH (p = 0.013) and control participants (p = 0.022). The average age of participants with incidental findings was 51.3 years and 49.1 years, versus 45.1 and 42.0 years without incidental findings in the PWH and control cohorts, respectively. There were no significant correlations with sex, or race/ethnicity (Table 4). The average CD4+ count in PWH with incidental findings was 714 cells/cumm, and for those without incidental findings 784 cells/cumm (p = 0.435). We found no correlation among presence or absence of incidental findings and HAND status for the cohort with HIV infection.

**Table 3:** Distribution of Findings in Participants with HIV by Age, Sex, Race/Ethnicity, CD4+ Level, and HAND status.

		No Incidental Findings Mean (SD) or n (%)	Incidental Findings Mean (SD) or n (%)	p-value
Age (in years)		45.1 (12.4)	51.3 (11.3)	0.013
**Sex**				
Male		43 (60)	20 (56)	0.679
Female		29 (40)	16 (44)	
**Race and Ethnicity**				
NH White Only		43 (60)	20 (56)	0.734
NH Black Only		19 (26)	12 (33)	
Other		10 (14)	4 (11)	
CD4+ (cells/μL)		784 (429)	714 (446)	0.435
Neurocognitive Disorder*				
Normal	48 (67)		20 (56)	0.429
ANI	12 (17)		11 (31)	
MND	7 (10)		3 (8)	
HAD	5 (7)		2 (6)	

*ANI = Asymptomatic Neurocognitive Impairment, MND = Mild neurocognitive disorder, HAD = HIV-associated Dementia

**Table 4:** Distribution of Findings in Control Participants by Age, Sex, and Race/Ethnicity.

	No Incidental Findings Mean (SD) or n (%)	Incidental Findings Mean (SD) or n (%)	p-value
Age (in years)	42.0 (14.2)	49.1 (16.8)	0.022
**Sex**			
Male	48 (52)	21 (64)	0.256
Female	44 (48)	12 (36)	
**Race and Ethnicity**			
NH White Only	60 (65)	16 (48)	0.205
NH Black Only	23 (25)	11 (33)	
Other	9 (10)	6 (18)	

## Discussion

We found high prevalence of incidental findings, but surprisingly, no significant difference in rates between the two groups with and without HIV infection. Increasing age was the only factor correlated with more frequent incidental findings. A smaller study comparing patients with HIV infection and demographically matched controls had similar findings, although in this study, the neuroradiologist was not blinded to the participant serostatus which may have led to some bias in reporting^3^.

In 1999, Katzman *et al*.^8^ retrospectively studied the prevalence of incidental imaging findings in a healthy asymptomatic population. This group reported an incidence of 18% incidental findings in their cohort of 1000 research subjects. At the other extreme, more recent studies show that incidental findings appear in 84% of populations with exposures to known neurotoxins^14^. In this study, we found a rate of 33.3% in the HIV cohort, 27% in the controls. Such a wide range of prevalence has been hypothesized to be due to varying methods, populations, and sample sizes^9,15,16^. Furthermore, there is evidence that the prevalence of incidental finding detection is more likely using high-resolution MRI sequences^17^. Certainly, as MRI technology continues to advance and achieve higher sensitivities and images achieve higher resolutions, we will likely continue to see increasing prevalence of incidental findings. For scientists conducting research using neuroimaging, the decision on how to manage disclosure of these findings will continue to pose a complex ethical dilemma^18^. Despite concerns raised by both clinical and non-clinical researchers, a consensus protocol for the management of incidental findings has not been reached^1,19^.

Chief among the ethical concerns posed by these findings is the necessity, timing, and context of disclosure to both patients and research participants. Generally, scientific research has followed the fundamental principle of *primum non nocere* (first, do no harm), as well as general duty to help and rescue^16^. The prevalence of clinically significant incidental findings among healthy participants is estimated around 2.7% in brain MRI, with a number needed to scan of around 37 for one finding deemed of clinical significance^15^. This study identified a spectrum of incidental findings ranging from benign sinus disease to brain neoplasms (Table 2), all of which have clinical significance to varying degrees. This further complicates the ethical debate surrounding disclosure, because the definitions of clinical significance are broad, the extent of potential harm by non-disclosure is often unknown, and general duty to help and rescue is often ill-defined and not-standardized. Risks of false-positive findings and the cost burden of further testing and imaging are important considerations.

Potential limitations of this study include the fact that matching did not include other potentially relevant factors such as cardiovascular disease risk factors, medication exposure, socioeconomic status, access to healthcare or lifestyle. Additionally, to maintain coherence with previous literature on incidental findings, abnormalities were classified according to a four-category scheme used in prior studies^4,5,14^. Thus, our system for classifying incidental findings was limited by the broad scope of the various categories which contained a spectrum of diagnoses that was broad in potential severity. Finally, as this is a post-hoc analysis, we did not plan to collect clinical outcomes of the incidental findings observed.

Although we found high levels of incidental findings in both arms, there was no statistically significant difference between the two groups. Age was the only factor correlated with rates of incidental findings in either PWH or controls. This is consistent with findings that have been previously reported in the general population and in patients with HIV infection^4,17^. Furthermore, in the cohort with HIV infection, there was no observable difference in the rates of incidental findings based on the presence or absence of HAND or degree of immunosuppression.

This work highlights and reinforces the need for consistent protocols for the handling of incidental findings on MRI scans in functional and structural brain research. The findings of this study will be relevant to future studies reliant upon brain imaging in PWH. Awareness of the high likelihood of incidental findings and knowledge that this risk increases with age can help researchers prepare for unexpected results and ultimately their clinical management during the consent process. Furthermore, research subjects will be more informed and can better prepare themselves for findings that do come up. Clinically, improved awareness of expected frequency and character of these potential findings can help clinicians better inform patients on the risks and benefits of undergoing a brain imaging study, both for research purposes and for clinical care.

## Data Availability

The authors will make data and associated protocols available to readers without undue qualifications in material transfer agreements.
